# Exploration of Macro-Micro Biomarkers for Dampness-Heat Syndrome Differentiation in Different Diseases

**DOI:** 10.1155/2013/706762

**Published:** 2013-04-21

**Authors:** Jianye Dai, Shujun Sun, Jinghua Peng, Huijuan Cao, Ningning Zheng, Junwei Fang, Qianhua Li, Jian Jiang, Yongyu Zhang, Yiyang Hu

**Affiliations:** ^1^Center for Traditional Chinese Medicine and Systems Biology, Shanghai University of Traditional Chinese Medicine, Shanghai 201203, China; ^2^Institute of Liver Diseases, Shuguang Hospital, Key Laboratory of Liver and Kidney Diseases of Ministry of Education, Shanghai University of Traditional Chinese Medicine, Shanghai 201203, China

## Abstract

Increased attention is being paid to traditional Chinese medicine (TCM) as a complementary and alternative medicine to provide an effective approach for personalized diagnosis and clinical treatment. TMC performs treatment based on differentiation of TCM syndrome (ZHENG), which may identify special phenotypes by symptoms and signs of patients even if they are in different diseases. There has, however, been skepticism and criticism because syndrome classification only depends on observation, knowledge, and clinical experience of TCM practitioners, which lacks objectivity and repeatability. In order to transform syndrome classification into mainstream medicine, we introduce a macro-micro approach that combines symptoms, clinical indicators, and metabolites. The present paper explores the macro-micro biomarkers of dampness-heat syndrome in chronic hepatitis B and nonalcoholic fatty liver patients, which could provide the basis for developing a possible population-screening tool for selecting target individuals and creating an evaluation index for personalized treatment.

## 1. Introduction

Chronic hepatitis B (CHB) and nonalcoholic fatty liver disease (NFL) are two common diseases occurring throughout the world that have continuously increasing morbidity [1]. It is worth noting that 12.1% [2] and 37.1% [3] of patients with CHB and NFL, respectively, exhibit the same symptoms (e.g., yellow and slimy fur), which are characteristics of dampness-heat syndrome (DH) in traditional Chinese medicine (TCM). Although CHB and NFL have different etiologies in Mainstream Medicine, TCM practitioners may perform the same treatment for these patients.

Actually, different diseases may be treated similarly in TCM particularly when the same syndrome appears in these diseases [4]. In this respect, syndrome differentiation and treatment (bian zheng lun zhi) may provide some new revelations to modern personalized medicine [5–7]. Syndrome differentiation is still debated, because it depends on clinical observation and TCM practitioners' experiences, which are thought to be subjective and unrepeatable. The success of personalized medicine relies on having accurate diagnostic tests that identify those patients who can benefit from targeted therapies [8]; thus, the ability to achieve objectivity and repeatability in TCM diagnosis would provide a greatly needed breakthrough.

Recently, researchers and scientists of TCM have explored incorporating several potentially beneficial methods, including, for example, physiology and biochemistry [9], molecular biology [10], and tongue image digitization [11, 12]. However, the classifications have been less than satisfactory. The main reason might be that these methods only focus on one or several indicators and thus cannot generalize the entire state of the syndrome. We therefore conceived the possibility of a macro-micro approach that includes a combination of metabolites, symptoms, and clinical indicators. Clinical manifestations are the macroeconomic performance, and metabolic molecules and indicators are microscopic. To serve in TCM diagnosis and treatment, here we report our findings from a case study that allowed us to preliminarily explore the macro-micro biomarkers of DH in CHB and NFL patients.

## 2. Experimental

### 2.1. Subjects and Experiment Design

Twenty healthy volunteers and 115 patients (60 patients for training and another 55 patients for testing) of dampness-heat syndrome chronic hepatitis B (DHHB), nondampness-heat syndrome chronic hepatitis B (NDHHB), and dampness-heat syndrome nonalcoholic fatty liver (DHFL) were enrolled in the study. The clinical study was approved by the local ethics committee and was performed in accordance with the principals contained in the Declaration of Helsinki. All individuals provided informed consent before inclusion into the study. Diagnostic standard of HB and FL patients was referred to “the guideline of prevention and treatment for chronic hepatitis B” [13] and “guidelines for management of nonalcoholic fatty liver disease: an updated and revised edition” [14]. Cases meeting the diagnostic criteria for chronic hepatitis B and nonalcoholic fatty liver, respectively, at 18–65 (39.9 mean ± 13.5 std. dev.) years of age who signed the informed consent form were included in the study. Individuals were excluded from the study if they met any of the following criteria. (1) Cases complicated with other hepatotropic virus hepatitis and alcoholic fatty liver. (2) Chronic severe hepatitis. (3) HB and FL patients associated with serious primary disease of heart, kidney, lung, endocrine, blood, metabolic and gastrointestinal, or psychotic patients. (4) Pregnant or lactating women. A junior medical physician made the initial diagnosis and recorded the information of four traditional examinations accurately and completely. Three more senior physician (either chief or deputy physicians) subsequently confirmed the initial diagnosis by the records and gave the hierarchical results of typical degree. Only those cases that were identified as classical DH patients by both the junior and the senior physicians were included in the study to guarantee the correctness of ZHENG differentiation.

### 2.2. Chemicals and Drugs

N,O-bis (trimethylsilyl) trifluoroacetamide (BSTFA with 1% TMCS) and urease were purchased from Sigma-Aldrich Co. LLC (USA). Methoxyamine hydrochloride, methanol, ethanol, myristic acid, chloroform and pyridine were purchased from China National Pharmaceutical Group Corporation (Shanghai, China).

### 2.3. Sample Collection and Preparation

A complete physical examination was given, and the health condition was recorded on a scale including the information obtained through four traditional examinations: looking, listening and smelling, asking, and touching when the patient entered the study. Seventy-one clinical indicators and 115 contents from the four methods of examinations were acquired for the basic information.

Urine samples were collected from all subjects and were stored at −80°C until GC-MS assay. All urine samples were thawed in an ice water bath and vortex-mixed before analysis. Each 1 mL aliquot of standard mixture or urine sample was placed into a screw top tube, samples were centrifuged for 10 min at (12,000 rpm), and 150 *μ*L supernatants were then transferred into clean screw top tubes. After adding 70 *μ*L of urease (4 mg/mL) and vortex-mixing for 30 s, samples were conditioned at 37°C for 15 min to remove the urea. After the addition of 800 *μ*L methanol and 10 *μ*L of myristic acid in methanol (1 mg/mL) and mixing for 1 min, the solution was centrifuged at 13,000 rpm for 10 min. A 200 *μ*L aliquot of supernatant was then transferred into a GC vial and evaporated to dryness under N_2_ at 30°C. Fifty *μ*L of methoxyamine in pyridine (15 mg/mL) was added to the GC vial, and vortex-mixed for 1 min, and the methoximation reaction was carried out for 90 min rocking in a shaker at 30°C, then 50 *μ*L of BSTFA plus 1% TMCS was added to the samples for trimethylsilylation for another 1 h at 70°C. In the final step, 30 *μ*L of heptane was added to the GC vial, and the solution was analyzed utilizing GC-MS after vortex for 30 s.

### 2.4. Data Acquisition

All GC-MS analyses were performed by a mass spectrometer 5975B (Agilent technologies, USA) coupled to an Agilent 6890 (Agilent technologies, USA) gas chromatography instrument. In the gas chromatographic system, a catabletary column (Agilent J&W DB-5 ms Ultra Inert 30 m × 0.25 mm, film thickness 0.25 *μ*m) was used. Helium carrier gas was used at a constant flow rate of 1.0 mL∗min⁡^−1^. One *μ*L of derivatized samples was injected into the GC/MS instrument, and splitless injection mode was used. A programmed column temperature was optimized to acquire a well separation. The temperatures of the injection port, the interface, and source temperature were set at 280°C, 260°C and 230°C, respectively. The measurements were made with electron impact ionization (70 eV) in the full scan mode (*m/z* 30–550). The solvent post time was set to 5 min. The GC-MS operating condition was the same as the previous experiment [15] except the column temperature program.

### 2.5. Data Analysis

Due to experimental variation and column aging, shifts in retention time between fingerprints may occur. When the total ion current chromatograms (TICs) were obtained, peak-alignment or warping techniques are commonly applied to compensate for minor shifts in retention times. Thus, in the subsequent data processing, the same variable manifested synchronous information in every profile. Therefore, all GC-MS raw files were converted to CDF format via the Agilent MSD Workstation software, and were subsequently processed by the XCMS toolbox (http://metlin.scripps.edu/download/) using XCMS's default settings with the following exceptions: xcmsSet (full width at half-maximum: fwhm = 5;  S/N cutoff value: snthresh = 10, max⁡ = 25), group (bw = 5). The resulting table (CSV file) was exported into Microsoft Excel (Microsoft Inc., USA) where normalization was performed prior to multivariate analyses. The resulting three-dimensional matrix involving peak index (RT-*m/z* pair), sample names (observations), and normalized peak area percent was introduced into Simca-P 11.5 Umetrics software (Umea, Sweden) that was used for analysis of principal component analysis (PCA), partial least squares discriminant analysis (PLS-DA), and orthogonal partial least squares (OPLSs). Differential variables with VIP values [16] exceeding 1.5 between two different groups were generated from OPLS loadings plot. Subsequently, those variables were further analyzed by Mann-Whitney *U*-test to confirm the changes in metabolites by SPSS 17.0 (SPSS, Chicago, IL, USA) with the threshold *P* value set at 0.1. Firstly, the variables were identified by searching in NIST 2005 database. Then, standard compounds were used to confirm some of the identified metabolites.

**Figure 1:**
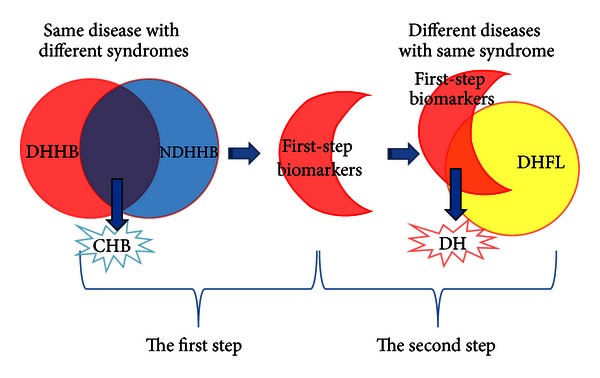
Schematic diagram of research approach for selection of DH.

Figure 1 shows a schematic diagram of the steps followed to determine the final list of potential biomarkers. The first step was to remove the differential information of CHB from the DHHB by removing the intersection of NDHHB and DHHB's differential information based on the ideas of the “same disease with different syndrome.” The reduced set of first-step biomarkers were further filtered by taking advantage of the “different diseases with same syndrome.” The final biomarkers were obtained from the intersection of the first-step DH biomarkers and biomarkers of DHFL.

## 3. Results

### 3.1. Establishment of the Potential Biomarkers of Clinical Symptoms and Indicators

**Figure 2:**
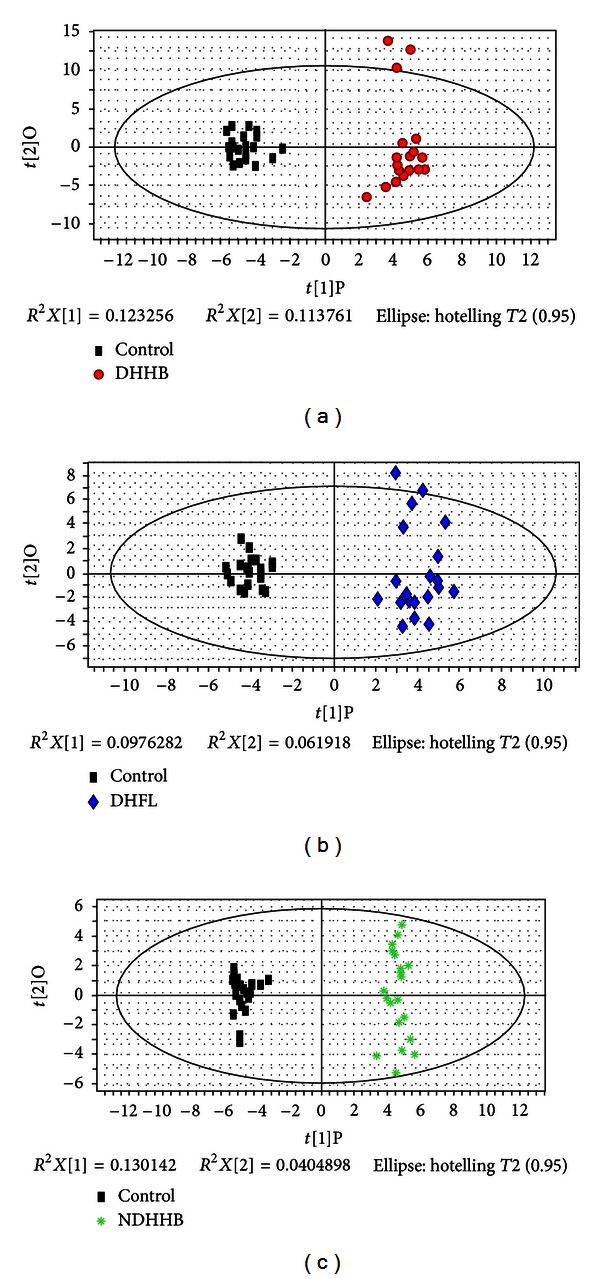
OPLS score plot of three syndromes compared to a healthy control group by symptoms and clinical indicators. (a) OPLS score plot of control and DHHB. (b) OPLS score plot of control and DHFL. (c) OPLS score plot of control and NDHHB.

All symptoms and clinical indicators were analyzed and utilized to distinguish the three syndrome groups (DHHB, NDHHB, and DHFL) and the healthy control group (control). Orthogonal partial least squares (OPLSs) was used to effectively extract variables responsible for the separation by removing variables unrelated to pathological status. Figures 2(a), 2(b), and 2(c) depict the OPLS score plots, which show that DHHB, NDHHB, and DHFL groups were clearly separated from the control group. The most meaningful characteristics were screened by OPLS loading plot analysis and are listed in Table 1. The quality of the model was characterized by two performance statistics, *R* ^2^ *Y* (cum) and *Q* ^2^ *Y* (cum), indicating the total explanation and predictability of the model [17]. The information of models is summarized in Table 4.

### 3.2. Establishment of the Potential Biomarkers of Urinary Metabolic Profiles

**Figure 3:**
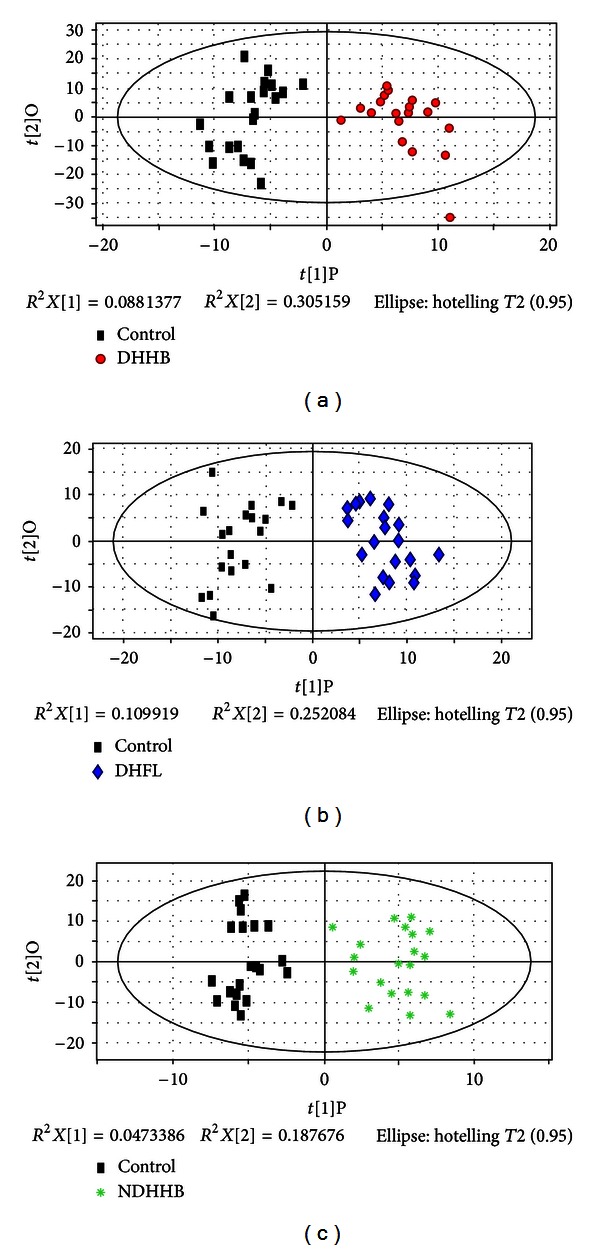
OPLS score plot of three syndromes compared to healthy control group by metabolites. (a) OPLS score plot of control and DHHB. (b) OPLS score plot of control and DHFL. (c) OPLS score plot of control and NDHHB.

Urine profiles obtained from GC-MS were analyzed for distinctions among the three syndromes and the control group by OPLS. Figures 3(a), 3(b) and 3(c) indicate the OPLS score plot, which show a clear separation for DHHB, NDHHB, and DHFL groups from the control group. The most important variables for the discriminative models were screened by loading plot analysis. The potential metabolic biomarkers of each syndrome differentiated from control group were identified by the NIST database and are summarized in Table 2. Model information is summarized in Table 4.

### 3.3. Establishment of Potential Biomarkers of DH in CHB and FL

Because groups of selected markers may contain information of syndrome and disease, the biomarkers of DH were further filtered. Thus the final set of potential biomarkers considered were those that remained after the intersection of DHHB and NDHHB was removed from DHHB, and were intersected with DHFL. Figure 1 shows a schematic diagram of the steps. As to the former works, the potential macro-micro biomarkers were obtained from the integration of differential metabolites, hierarchical corresponding symptoms and clinical indicators. The potential biomarkers are listed in Table 3.

### 3.4. Preliminary Verification of Identified Biomarkers

**Figure 4:**
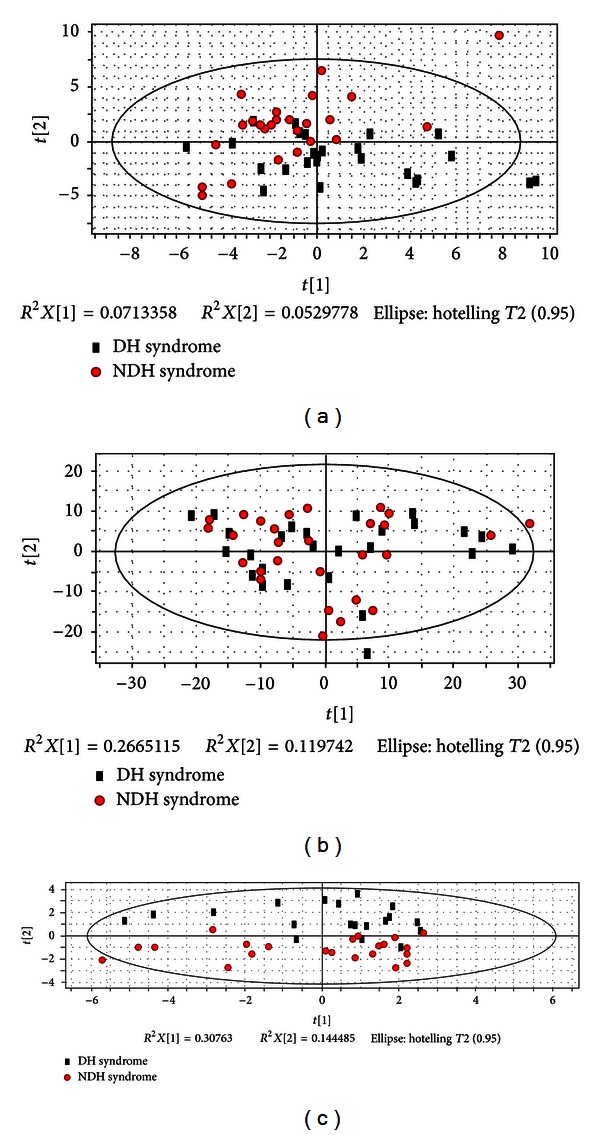
PCA score plot of DH versus NDH with potential biomarkers. (a) PCA score plot of DH and NDH only by symptoms and clinical indicators. (b) PCA score plot of DH and NDH only by metabolites. (c) PCA score plot of DH and NDH by macro-micro biomarkers from the integration of differential metabolites, hierarchical corresponding symptoms and clinical indicators.

The potential biomarkers were verified in 55 blind test cases of CHB with two Syndromes (Dampness-Heat Syndrome (DH) and Non-Dampness-Heat Syndrome (NDH)) for Syndrome classification. Using only the potential biomarkers or only the clinical symptoms and indicators did not differentiate the two syndromes satisfactorily (Figures 4(a) and 4(b)); however, by including metabolites, symptoms, and clinical indicators in the analysis, resulted in a stronger differentiation (Figure 4(c)). It is worth mentioning that former classifications (in Sections 3.1 and 3.2) were performed by supervised OPLS, owing to the complexity of clinical samples. However, the DH could be classified from NDH by unsupervised PCA in this verification with the selected biomarkers, which revealed the strong ability of DH differentiation, though they need further verification in clinical.

## 4. Discussion

In this study, we attempted to explore the macro-micro biomarkers of DH, which could provide the feasibility and robustness for syndrome differentiation. The selected metabolites of DH were considered to be related with the pathogenesis. By analysis of KEGG (http://www.genome.jp/kegg/), the 11 metabolic markers are related to biosynthesis of secondary metabolism, microbial metabolism in diverse environments, carbon fixation pathway in prokaryote, proteins digestion and absorption, and carbohydrate digestion and absorption, which could be classified in microbial metabolism and digestive capacity. These may correspond with “the disorder in transportation and transformation of the essence from food and drink” in TCM, which was regarded as one important reason for Dampness-Heat Syndrome [18, 19].

Aspartate transaminase (AST) is the only clinical indicator in our biomarkers. It may suggest that the clinical indicators are limited to classify the syndromes. But AST has been reported to be connected to DH, with odds ratio (OR) value equal to 5.49 [20]. There is thus strong evidence that DH reflects inflammation of the liver damage.

Tongue diagnosis is of great importance for syndrome differentiation in TCM, determining the treating principle, prescribing a formula, and predicting the prognosis [21]. Except string-like pulse, other differential symptoms are the characterization of tongue, which is one of the direct objective bases for TCM clinical diagnosis and treatment. In our opinion, pulse diagnosis is as important as tongue diagnosis, so ultimately a more comprehensive analysis for the combination of them is needed.

Although only metabonomics was utilized in this study, we suggest that it would be valuable to expand beyond Metabonomics to system biology owing to the similarity between the various omics. Including a full System Biology approach to the determination of informative biomarkers will provide a more comprehensive and accurate syndrome differentiation. We thus suggest that genes, proteins, metabolites, and clinical information should all be integrated in future analyses.

## 5. Conclusion

This study is the first time that biomarkers of DH were obtained by a macro-micro approach with the integration of omic and clinical information to provide an effective and objective and repeatable approach for Chinese personalized medicine. Moreover, the preliminary verification indicated the feasibility and robustness of the approach for dampness-heat syndrome differentiation. Thus, these DH biomarkers could be used to provide a foundation on which we can to develop a possible population-screening tool for selecting target individuals and for creating an evaluation index for personalized treatment based on syndrome differentiation.

## Figures and Tables

**Table 1:** Significantly different symptoms and clinical indicators identified in the three syndromes compared to a healthy control group.

Indicators and symptoms	Group	VIP^a^	*P*(M-W)^b^	FN^c^
Alkaline phosphatase	DHHB	1.69	0.00	+2.03
Apolipoprotein A-1	DHHB	1.74	0.00	+1.91
Aspartate aminotransferase	DHHB	1.62	0.00	+2.77
Glutamyltransferase	DHHB	1.65	0.00	+2.06
Immunoglobulin G	DHHB	2.01	0.00	+2.06
Prealbumin	DHHB	2.8	0.00	−2.56
*β*-globin	DHHB	2.52	0.00	+2.67
Thick fur	DHHB	1.81	0.00	+1.73
Bitter taste	DHHB	1.79	0.00	+1.89
Slimy and curdy fur	DHHB	1.75	0.00	−1.73
Mean corpuscular hemoglobin concentration	DHHB	1.84	0.00	+2.15
Tongue color	DHHB	2.15	0.00	+1.96
Basophil	DHHB	2.07	0.00	+2.56
Fur color	DHHB	2.68	0.00	+2.58
String-like pulse	DHHB	1.81	0.00	+1.73
Alanine aminotransferase	DHFL	2.08	0.00	+2.16
Aspartate aminotransferase	DHFL	1.78	0.00	+1.82
Total cholesterol	DHFL	2.10	0.00	+1.87
Triglyceride	DHFL	2.44	0.00	+2.60
*β*-globin	DHFL	2.54	0.00	+2.31
Thick fur	DHFL	1.92	0.00	+1.64
Lack of strength	DHFL	2.11	0.00	+1.64
Dysphoria	DHFL	1.98	0.00	+1.67
Slimy and curdy fur	DHFL	2.58	0.00	−2.03
Uric acid	DHFL	2.45	0.00	+2.31
Glucose	DHFL	1.95	0.00	+1.92
Tongue color	DHFL	2.03	0.00	+1.68
Systolic pressure	DHFL	1.89	0.00	+2.02
Diastolic pressure	DHFL	2.29	0.00	+2.37
Fur color	DHFL	3.08	0.00	+2.58
Weight	DHFL	2.79	0.00	+2.44
String-like pulse	DHFL	1.92	0.00	+1.64
Alkaline phosphatase	NDHHB	1.61	0.00	+1.87
Apolipoprotein A-1	NDHHB	2.07	0.00	+2.39
Activated partial thromboplastin time	NDHHB	1.60	0.00	+1.68
Hepatitis B core antibody	NDHHB	2.85	0.00	+2.90
Hepatitis B core antibody-immunoglobulin M	NDHHB	1.76	0.00	+2.90
Hepatitis B surface antigen	NDHHB	3.04	0.00	+2.90
Immunoglobulin G	NDHHB	1.60	0.00	+1.97
Prealbumin	NDHHB	2.38	0.00	−2.28
Triglyceride	NDHHB	1.77	0.00	+1.85
Total protein	NDHHB	1.85	0.00	+2.01
*β*-globin	NDHHB	2.54	0.00	+2.79
Teeth-marked tongue	NDHHB	2.11	0.00	+1.92
Gallbladder	NDHHB	2.04	0.00	+1.92
Relaxed pulse	NDHHB	1.69	0.00	+1.56
Lack of strength	NDHHB	1.93	0.00	+1.73
Mean corpuscular hemoglobin concentration	NDHHB	1.79	0.00	+2.11
Pre-S1 antibodies	NDHHB	3.14	0.00	+2.90
Pre-S1 antigen	NDHHB	3.14	0.00	+2.90
Luxuriant or withered tongue	NDHHB	1.73	0.00	+1.73
Soggy pulse	NDHHB	1.93	0.00	+1.73
Basophil	NDHHB	2.34	0.00	+2.41
Diastolic pressure	NDHHB	1.56	0.00	+1.72
Mean platelet volume	NDHHB	2.30	0.00	+2.58

^a^VIP: variable importance in the project.

^b^ *P*(M-W) value was obtained from Mann-Whitney test (syndromes compared to healthy control).

^c^FN is fold change of mean ranks calculated by the Mann-Whitney test (syndromes compared to healthy control). “+” means upregulated and “−” means downregulated.

**Table 2:** Significantly different metabolites identified in the three syndromes compared to a healthy control group.

Compound	Group	VIP^a^	*P*(M-W)^b^	FN^c^
Acetic acid*	DHHB	1.87	0.00	−2.07
Succinic acid*	DHHB	1.85	0.01	−1.61
D-Xylose*	DHHB	2.22	0.00	−1.96
Maltose	DHHB	1.92	0.00	+1.89
Butyrate*	DHHB	1.87	0.00	−1.72
Aminolevulinic acid*	DHHB	1.67	0.00	−1.75
Ribitol	DHHB	2.22	0.00	−1.93
Creatinine*	DHHB	1.77	0.01	−1.63
Benzene*	DHHB	1.88	0.01	−1.56
2-Butenoic acid*	DHHB	1.81	0.02	−1.53
(R)-Mandelic acid*	DHHB	1.68	0.01	−1.59
Glutaconic acid*	DHHB	1.68	0.02	−1.54
Tartronic acid	DHHB	2.28	0.00	−2.10
Benzophenone	DHHB	1.58	0.02	−1.53
Pteridine	DHHB	1.75	0.00	−1.71
3-Amino-1,2,4-triazole	DHHB	1.74	0.02	−1.51
3-Indole butanoic acid*	DHHB	1.61	0.02	−1.50
1-Cyclohexene carboxylic acid	DHHB	1.93	0.01	−1.66
3-Indole acetic acid*	DHHB	1.58	0.00	−1.73
Pyrazinoic acid*	DHHB	1.62	0.00	−2.00
Acetic acid*	DHFL	1.52	0.00	−1.70
Succinic acid*	DHFL	1.90	0.00	−1.81
D-Fructose	DHFL	1.58	0.00	−1.75
D-Galactose	DHFL	1.69	0.00	−1.99
Benzoic acid	DHFL	1.51	0.01	−1.59
Butyrate*	DHFL	1.54	0.01	−1.65
D-Gluconic acid	DHFL	1.51	0.00	−1.70
Amino levulinic acid*	DHFL	1.54	0.00	−1.82
Glutarate	DHFL	1.57	0.01	−1.61
Creatinine*	DHFL	1.65	0.00	−1.69
Gulonic acid	DHFL	1.65	0.00	−1.72
Glucaric acid	DHFL	1.63	0.00	−2.02
3-Indole acetic acid*	DHFL	1.72	0.00	−2.09
(R)-Mandelic acid*	DHFL	1.76	0.00	−1.75
3-Indole butanoic acid*	DHFL	1.68	0.00	−1.68
1-Cyclohexenecarboxylic acid	DHFL	1.86	0.00	−1.81
Pseudouridine	DHFL	1.79	0.00	−2.10
Glutaconic acid*	DHFL	1.77	0.00	−1.71
Tetradecanoic acid	DHFL	1.68	0.00	−1.74
Ethylene	DHFL	1.51	0.01	−1.57
Pteridine	DHFL	1.57	0.00	−1.73
Pyrazinoic acid*	DHFL	1.53	0.00	−2.19
1-(1-Benzyl-1H-indol-3-yl)-	DHFL	1.63	0.00	−1.83
2,2,2-trifluoroethanone				
Glycine*	NDHHB	2.11	0.01	+1.62
D-Xylose*	NDHHB	2.15	0.01	−1.57
D-Gluconic acid	NDHHB	1.87	0.00	+1.85
Ribitol	NDHHB	2.19	0.01	−1.57
2,3-Butanedione	NDHHB	1.89	0.02	+1.56
Tartronic acid	NDHHB	2.21	0.00	−1.66
Vanillylmandelic acid	NDHHB	1.94	0.05	+1.42
3-Amino-1,2,4-triazole	NDHHB	2.31	0.03	−1.48

^a^VIP: variable importance in the project.

^b^ *P*(M-W) value was obtained from Mann-Whitney test (syndromes compared to healthy control).

^c^FN is fold change of mean ranks calculated by the Mann-Whitney test (syndromes compared to healthy control). “+” means upregulated and “−” means downregulated.

*These metabolites were identified by NIST library and standards; others were only identified by NIST library.

**Table 3:** List of the macro-micro biomarkers of DH in CHB and NFL.

Biomarkers	Category
(R)-mandelic acid	Metabolites
1-Cyclohexenecarboxylic acid	Metabolites
3-Indole acetic acid	Metabolites
3-Indole butanoic acid	Metabolites
Acetic acid	Metabolites
Amino levulinic acid	Metabolites
Butyrate	Metabolites
Creatinine	Metabolites
Glutaconic acid	Metabolites
Pteridine	Metabolites
Pyrazinoic acid	Metabolites
Succinic acid	Metabolites
Aspartate aminotransferase	Indicators
Thick fur	Symptoms
Slimy and curdy fur	Symptoms
Tongue color	Symptoms
Fur color	Symptoms
String-like pulse	Symptoms

**Table 4:** Summary of the modeling quality of OPLS analysis.

Name	No^a^	*R* ^2^ *X*_cum_^b^	*R* ^2^ *Y*_cum_^c^	*Q* ^2^ *Y*_cum_^d^
1A	1P + 1O^e^	0.24	0.97	0.91
1B	1P + 1O	0.16	0.96	0.78
1C	1P + 1O	0.17	0.98	0.93
2A	1P + 2O	0.50	0.89	0.70
2B	1P + 3O	0.56	0.90	0.48
2C	1P + 3O	0.49	0.91	0.57

^a^No represents the number of components.

^b,c^ *R* ^2^ *X*_cum_ and *R* ^2^ *Y*_cum_ represent the cumulative sum of squares (SSs) of all the *X*'s and *Y*'s explained by all extracted components.

^d^ *Q* ^2^ *Y*_cum_ is an estimate of how well the model predicts the *Y*'s.

^e^1P + 1O: one predictive component and one orthogonal component for establishing the OPLS model.
